# Use of a Conditional *Ubr5* Mutant Allele to Investigate the Role of an N-End Rule Ubiquitin-Protein Ligase in Hedgehog Signalling and Embryonic Limb Development

**DOI:** 10.1371/journal.pone.0157079

**Published:** 2016-06-14

**Authors:** Elaine Kinsella, Natalie Dora, David Mellis, Laura Lettice, Paul Deveney, Robert Hill, Mark Ditzel

**Affiliations:** 1 Edinburgh CRUK Cancer Research Centre, MRC Institute of Genetics and Molecular Medicine at the University of Edinburgh, Western General Hospital, Edinburgh, UK; 2 MRC Human Genetics Unit, MRC Institute of Genetics and Molecular Medicine at the University of Edinburgh, Western General Hospital, Edinburgh, UK; Laboratoire de Biologie du Développement de Villefranche-sur-Mer, FRANCE

## Abstract

Hedgehog (Hh) signalling is a potent regulator of cell fate and function. While much is known about the events within a Hh-stimulated cell, far less is known about the regulation of Hh-ligand production. *Drosophila* Hyperplastic Discs (Hyd), a ubiquitin-protein ligase, represents one of the few non-transcription factors that independently regulates both *hh* mRNA expression and pathway activity. Using a murine embryonic stem cell system, we revealed that shRNAi of the mammalian homologue of *hyd*, *Ubr5*, effectively prevented retinoic-acid-induced *Sonic hedgehog* (Shh) expression. We next investigated the UBR5:Hh signalling relationship in vivo by generating and validating a mouse bearing a conditional *Ubr5* loss-of-function allele. Conditionally deleting *Ubr5* in the early embryonic limb-bud mesenchyme resulted in a transient decrease in *Indian hedgehog* ligand expression and decreased Hh pathway activity, around E13.5. Although *Ubr5*-deficient limbs and digits were, on average, shorter than control limbs, the effects were not statistically significant. Hence, while loss of UBR5 perturbed Hedgehog signalling in the developing limb, there were no obvious morphological defects. In summary, we report the first conditional *Ubr5* mutant mouse and provide evidence for a role for UBR5 in influencing Hh signalling, but are uncertain to whether the effects on Hedgehog signaling were direct (cell autonomous) or indirect (non-cell-autonomous). Elaboration of the cellular/molecular mechanism(s) involved may help our understanding on diseases and developmental disorders associated with aberrant Hh signalling.

## Introduction

In multicellular organisms Hedgehog (Hh) morphogens play an essential role in tissue/organ development [1] and their subsequent maintenance [2]. Acting as an extracellular signalling molecule, Hh ligands signal in a predominantly paracrine manner to convey information to neighbouring cells. Tight regulation of Hh ligand expression ensures temporal-spatial generation of morphogen gradients, which in turn ensure a well co-ordinated and appropriate cellular response [3]. The importance of correct Hh expression patterns is clear from the numerous human diseases (e.g., gastrointestinal, pancreatic and skin cancers) [4] and developmental disorders (e.g., cyclopia, cleft lip and limb abnormalities) [5] that result from its misexpression.

Mammals express three Hh ligands (Sonic-, Indian and Desert-Hedgehog) that exhibit distinct expression patterns throughout the body (EMBL Expression Atlas). Engagement of Hh ligands with one of their receptors Patched (Ptch1) [6–8] results in derepression of the Hedgehog signal transduction pathway, activation of the GLI family zinc finger (GLI) family of transcription factors and the subsequent transcription of GLI target genes. Activation of the Hh pathway (HhP) influences a wide range of cellular responses that include promotion of proliferation, differentiation and suppression of apoptosis[1]. Hedgehog signalling affects cell behaviour in multiple tissues and is heavily implicated in the communication between cells, including adult stem cells and their niches[2].

A large body of work has focused on investigating *Sonic hedgehog* (*Shh*) expression and function during animal development, with a particular focus on the developing limb[9]. Within the embryonic limb bud, *Shh* expression is spatially restricted to a posteriorly located zone of polarising activity (ZPA)[10], where its expression is regulated by a long-range enhancer element called the zone of polarizing activity regulatory sequence (ZRS) [11]. SHH expression within the ZPA, and its subsequent diffusion/transport across the tissue, sets up a posterior-anterior morphogen gradient that acts to instruct distinct cell fates and govern digit formation [9]. A number of human disease-associated point mutations within, or chromosomal translocations affecting, *Shh’s* regulatory regions underlie deregulated *SHH/Shh* expression and digit abnormalities [12].

While the molecular details concerning *Shh* DNA regulatory elements are well-described [13], far less is known about the proteins and upstream signalling pathway(s) that regulate *Shh* expression. In mammals Ras-associated signalling appears to promote *Shh* expression [14], while in *Drosophila*, Hyperplastic Disc (Hyd) suppresses *hh* ligand expression [15, 16]. Mechanistically, Hyd appears to regulate *hh* expression through influencing Shaggy–the *Drosophila* ortholog of Glycogen synthase kinase β (GSK3β) [16]. The ability of Hyd and its human ortholog E3 identified by Differential Display (EDD) to bind GLI2 [16], one of the HhP’s major transcriptional effectors, potentially places Hyd/EDD both upstream and downstream of the Hedgehog ligand activity. Here, we addressed whether Hyd’s murine homologue Ubiquitin Protein Ligase E3 Component N-recognin 5 (UBR5) could also influence Hedgehog pathway activity and ligand expression.

UBR5 contains a number of domains related to ubiquitin signalling, which include a Ubiquitin binding domain (UBA) [17], a substrate recruitment domain for N-end rule substrates called the Ubiquitin-protein ligase E3 component N-Recognin (UBR) domain [18, 19] and a catalytic Homologous to E6AP C-terminus (HECT) domain [20]–the presence of which defines UBR5 as an E3 ubiquitin-protein ligase. Functionally, UBR5 has previously been linked to DNA damage signalling [21–23], miRNA activity [24], metabolism [25] and cell cycle checkpoint control [26–30].

Our data supports a potential role for UBR5 in influencing *hedgehog* family member ligand expression and HhP activity, although we are unclear about the mechanism.

## Materials and Methods

### mES 14 cell culture and retinoic acid treatment

E14 mouse embryonic stem (mES) cells were cultured on 0.1% porcine gelatin (Sigma) coated 6-well plates at 37°C and 5% CO_2_ in GMEM (Invitrogen) supplemented with 10% foetal bovine serum, 10^3^ U/ml leukaemia inhibitory factor (Millipore). Retinoic acid (Sigma) or DMSO (Sigma) vehicle was added to the media and refreshed every 24 hours.

### shRNAi transfection and selection

6-well plates containing 2x10^6^ cells in antibiotic-free GMEM were transfected with 4μg of *pLKO*-based, puromycin-expressing, *Ubr5* shRNAi constructs (Sigma MISSION, TRCN0000003411 (*Ubr5*.*1*), TRCN0000226458 (*Ubr5*.*2*) and TRCN0000238587 (*Ubr5*.*3*) with Effectene (Qiagen) as per the manufacturer’s instruction. 24 hours later, transfected cells were selected in 0.1μg/ml puromycin (Sigma) and resistant colonies were pooled.

### RNA extraction, reverse transcription and PCR

RNA was extracted from embryos, dissected limb buds or ES cells using QIAshredder homogenisers coupled with RNeasy kits (QIAGEN) as per the manufacturers instructions. cDNA was produced using a First Strand cDNA Synthesis Kit also as per manufacturer’s instructions (Roche). All semi-quantitative RT-PCR was carried out using Platinum Taq Polymerase PCR kit (Invitrogen) using a MJ Research Tetrad PTC-225 PCR machine. Primers used: *Shh* For GCC TAC AAG CAG TTT ATT CCC AAC and Rev CAG TGG ATG TGA GCT TTG GAT TC; *Ubr5* For CTC GAG GAA AGC TAG AGC AAA AAA TAA AAA GCC CAA ATC CAG and Rev GAG CTC TAC AGC GAC ATA GGC ACC ATC TAC C; *β-actin* For GGC CCA GAG CAA GAG AGG TAT CC and Rev ACG CAC GAT TTC CCT CTC AGC; *Nanog* For ACC TGA GCT ATA AGC AGG TTA AGA C and Rev GTG CTG AGC CCT TCT GAA TCA GAC; *Bmp4* For GAG GAG TTT CCA TCA CGA AGA and REV GCT CTG CCG AGG AGA TCA; *Klf4* For CCA GCA AGT CAG CTT GTG AA and Rev GGG CAT GTT CAA GTT GGA TT. Quantitative RT-PCR (qRT-PCR) was carried out using the Roche Universal probe Library coupled with the Lightcycler®480 system as per manufacturer’s instructions (Roche). Gene specific assays were designed using the online Assay Design Centre Tool (www.universalprobelibrary.com): *Shh* For ACC CCG ACA TCA TAT TTA AGG A and Rev TTA ACT TGT CTT TGC ACC TCT GA (UPL probe 32); *Ubr5* For TCA GCT CGA AGA GAG AGG ATG and Rev GCT CAG CAA TGT AGC ACG TC (UPL probe 103). The β-actin control reagent master mix was used to generate an internal reference for all reactions. Relative expression levels were determined using the ΔCt model [31].

### SDS-PAGE and Western blotting

Cells and dissected limb buds were lysed in a 1%TX-100 lysis buffer and processed as described previously [32]. For the limb buds, cytoplasmic and nuclear fractions were separated using NE-PER® nuclear and cytoplasmic protein extraction kit (Thermo Scientific). Proteins (30μg) were resolved by SDS-PAGE using 3–8% Tris-acetate gradient gels with Tris-acetate Running Buffer (Invitrogen) and wet-blotted onto PVDF membrane (Millipore) overnight at 4°C using Towbin buffer. Membranes were blocked in 5% milk/PBS at room temperature (RT) for one hour and incubated with primary antibodies against: UBR5 (Santa Cruz goat EDD M-19 1:2500); β-tubulin (Sigma mouse 1:30,000) and HP-1 (Chemicon mouse 1:10,000) for one hour at RT in 0.1% Tween PBS (PBST). Membranes were then washed three times in PBST at RT, incubated with appropriate secondary antibodies: donkey αgoat-HRP and horse αmouse-HRP, both (Jackson Labs 1:10,000) and washed three times in PBST at RT. Membranes were then incubated in ECL solution and imaged using the digital ChemiDoc imaging system (Promega).

### In situ hybridisation

*Shh* and *Ptch1* vectors and probes were previously described [11]. Primers incorporating restriction enzymes sites were used to amplify the probe from full-length cDNA template temples. Products were cloned into Bluescript SK and KS for antisense and control sense probe production. Riboprobes were generated from linearised vector templates using the MEGAscript kit (Life technologies) as per the manufacturer’s instruction. Embryos were dissected and fixed in 4% paraformaldehyde (Sigma) overnight at 4°C. In situ hybridisation was carried out as previously described [11]. Expression patterns were detected by the alkaline phosphatase substrates nitro-blue tetrazolium chloride (NBT) and 5-bromo- 4-chloro-3'-indolyphosphate p-toluidine (BCIP) (Roche).

### General animal work

Animal studies were approved by Medical Research Council Institute of Genetics and Molecular Medicine ‘Animal Care and Use Committee’ (applications PL21–06 and PL26–11) and carried out according to guidance issued by the Medical Research Council in Responsibility in the Use of Animals for Medical Research (July 1993), EU Directive 2010 and UK Home Office Project License no PPL 60/4424. Mice had constant access to food and water and were maintained on 12 hour light and dark cycles. Pups were weaned at three weeks old at which point ear clips were collected for genotype analysis. Timed matings were set up with E0.5 (embryonic day) taken as the morning a vaginal plug was detected. Following Schedule 1 sacrifice of the mother (CO_2_ asphyxiation followed by cervical dislocation), embryos were dissected out using a Leica EZ4HD dissecting microscope. Genomic DNA was extracted either from adult mouse earclips or embryo yolk sacs and analysed using the HotShot DNA extraction technique [33] using Platinum Taq (Invitrogen) according to the manufacturer’s instruction. Primers used were: Wild type *Ubr5* For 5’ GTT TCT GGC AAG GTT CAG TGC; Rev 5’ CAC ACA TGC TGC ACA AAC ACA TG; Ubr5mt* For 5’ GTT TCT GGC AAG GTT CAG TGC; Ubr5mt* Rev 5’ GCC ACT ATG CGC ACA GCT GG; *Ubr5*WT* For 5’ CGC GAA GAG TTT GTC CTC AC; Ubr5WT* Rev 5’ GCC TCG ATC CTC CCT TTA TC; *Neomycin* For 5’ TGT TCC GGC TGT CAG CGC AG; *Neomycin* Rev 5’ GAT ATT CGG CAA GCA GGC ATC; *FLP* For 5’ AGG GTG AAA GCA TCT GGG AGA; *FLP* Rev 5’ TCA ACT CCG TTA GGC CCT TCA; *Cre* For 5’ GCA TTA CCG GTC GAT GCA ACG AGT GAT GAG; *Cre* Rev 5’ GAG TGA ACG AAC CTG GTC GAA A.

### Creation of the conditional *Ubr5*^*mt*^ mouse

E14 embryonic stem (ES) cells carrying the *Ubr5* gene trap EUCE0171f01 were obtained from EUCOMM (European Conditional Mouse Mutagenesis Program). Chimeric mice were generated by injection of E14 mouse ES cells positive for the gene trap into C57BL/6J ES blastocysts that were transferred to pseudopregnant C57BL/6J females. Germline offspring were identified by coat colour and PCR genotyping confirmed the presence of the modified allele. Mice were then crossed to, and subsequently maintained on, a C57BL/6J background.

### Conditional disruption of *Ubr5* expression

For conditional studies, mice were first crossed to a mouse line expressing an enhanced form of Flippase (Flp-e) [34]. The presence of the inverted genetrap (*Ubr5*^WT*^) was confirmed by PCR. *Ubr5* expression was conditionally ablated in the whole embryo or limb buds by crossing *Ubr5*^WT*^ with *pCAGG-Cre-ER*^*T2*^[35] or *Prx1-Cre* [36] mice, respectively. Animal stocks were maintained as heterozygotes. Crosses were carried out using heterozygous animals to permit littermate controls and *Cre* was passed through the male germline. For viability studies, pregnant females underwent intraperitoneal (i.p.) injections of 4mg Tamoxifen (Sigma, stock 20mg/ml) at 11.5 days post coitum (dpc), followed by embryo collection at E13.5, E15.5 or E17.5.

### X-gal staining

X-gal staining was carried out as previously described [11]. Briefly, embryos were dissected out, fixed in 4% PFA at 4°C, washed and stained overnight. Stained embryos were then imaged whole mount and then sectioned for histology.

### Embryo embedding, sectioning and histological processing

Embryos were hand embedded in paraffin wax as per established in-house protocols. Wax blocks were allowed to solidify for three hours on a cold block, then mounted on wooden blocks and 4–8μm sections cut using a microtome (Leica). Sections were floated on a 45°C water bath prior to mounting on SuperFrost® slides (Fisher) which were left to dry overnight at 37°C prior to staining. Sections were de-waxed and stained using standard histological techniques for haematoxylin, alcian blue and alizarin (Sigma) staining.

### Macroscopy

Colour brightfield imaging of all embryos and embryo limbs was carried out on a Nikon AZ100 macroscope attached to a QImaging Micropublisher. Images were captured using IPLab software (Scanalytics). Histological sections were scanned using a Nanozoomer slide scanner (Hamamatsu) and visualised using NDPview2 software (Hamamatsu).

### Optical Projection Tomography (OPT)

OPT was carried out as previously described [37]. Briefly, embryos were fixed overnight using 4% paraformaldehyde, transferred to PBS and embedded in 1% low melting point molten agarose, dehydrated overnight and then cleared in a 2:1 mixture of benzyl alcohol to benzyl benzoate. Sample autofluorescence was analysed using a Bioptonics 3001 OPT scanner and reconstructed using Bioptonics view software.

### Statistics and computer programmes

Microsoft Excel and GraphPad Prism were used for graphs and the indicated ANOVA and t-test statistical analysis. Graphs indicate p values with the following key: *** = ≤0.0001; *** = ≤0.001; ** = ≤0.01; * = ≤0.05 and ns = ≥0.05. Adobe Photoshop CS6 and Illustrator CS6 were used to produce the figures.

## Results

### Retinoic acid promotes *Shh* expression in murine ES cells

**Fig 1:**
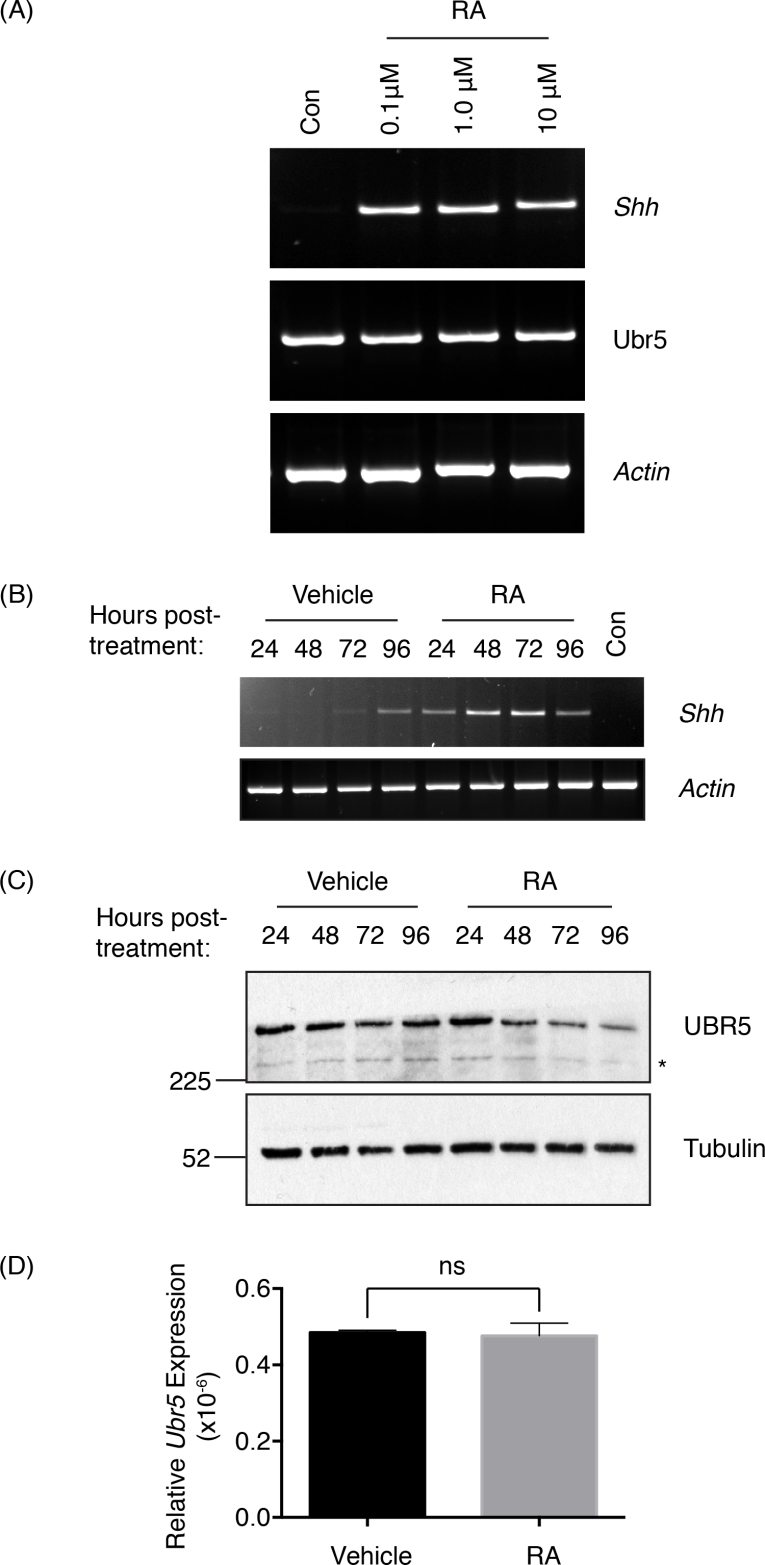
Retinoic Acid induces *Shh* and suppresses UBR5 expression. Murine E14 ES cells were treated with (A) the indicated concentration of RA, or (B-D) 0.1μM RA or DMSO (Vehicle) and analysed by RT-PCR at (A) 24hrs and (B) at the indicated times post-RA-treatment. (C) SDS-PAGE and Western blotting determined UBR5 expression over the indicated RA time course. An asterisk denotes an uncharacterised, faster migrating UBR5 antibody reactive species. Tubulin was used as a loading control. (D) qRT-PCR of *Ubr5* expression normalised against *β-actin* in mES cells 96hrs post-RA-treatment (n = 3, s.e.m indicated). Statistical analysis by Students t-test. ns = not significant.

Based upon the ability of Hyd, the *Drosophila* orthologue of UBR5, to influence *hh* ligand expression we wished to examine whether murine UBR5 was capable of regulating *Shh* expression. We chose to use an E14 mouse embryonic stem (mES) cell system that utilises retinoic acid (RA) treatment to induce *Shh* expression[38]. In contrast to Okada et al, we utilised conditions to restrain embryoid body formation and ES cell differentiation (i.e., retention of LIF[39] and 10% FBS[40] in the ES cell media throughout). Initially we confirmed that RA stimulation promoted mES cells to express *Shh* (Fig 1A). 24 hrs post-RA-treatment, *Shh* levels were readily detectable by RT-PCR, with doses as low as 0.1μM RA inducing significant *Shh* expression [41]. More detailed analysis of the inductive response with 0.1μM RA revealed peak *Shh* expression 48–72hrs after treatment (Fig 1B). At 96 hrs post-treatment, an increase in *Shh* expression was also detected in vehicle (DMSO) treated cells, potentially reflecting a possible effect of increased cell confluence.

Analysis of UBR5 protein expression in RA-treated cells revealed a significant reduction at 48hrs post-treatment (Fig 1C), which was sustained up to the end of the time course at 96hrs. To address if the loss of UBR5 expression 96hrs post-RA-treatment was due to transcriptional changes we used qRT-PCR analysis, which revealed no significant reduction (Fig 1D). These results suggested that the reduction in UBR5 expression was potentially via a post-transcriptional mechanism. Intriguingly, maximal *Shh* expression (48–72hrs post-RA-treatment) coincided with the marked reduction in UBR5 expression. However, at 24hrs post-RA-treatment, at a time *Shh* was initially induced (Fig 1B), UBR5 expression was not reduced (Fig 1C). In summary, it appeared that reduced UBR5 protein levels did not coincide with the initial induction of *Shh*, but did coincide with the subsequent increase in *Shh* expression level.

### UBR5 promotes RA-mediated *Shh* expression

**Fig 2:**
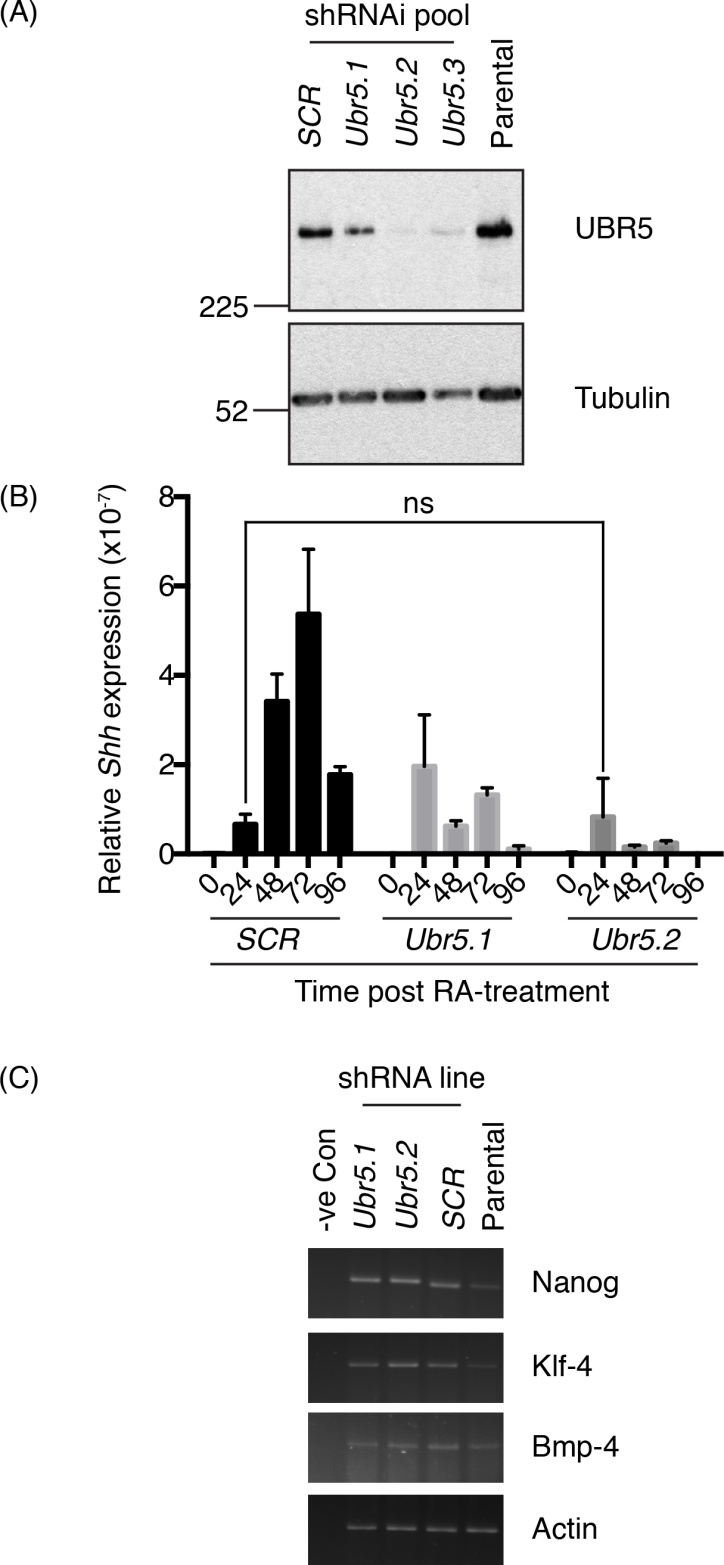
UBR5 is required for RA-mediated induction of *Shh*. Pools of murine E14 mES cells expressing either scrambled control (SCR) or *Ubr5* shRNAi were tested for (A) UBR5 expression by SDS-PAGE and Western blotting, using Tubulin as a loading control; (B) RA-mediated *Shh* induction by qRT-PCR (n = 3, relative expression levels to β-actin, s.e.m indicated) or (C) expression analysis of the indicated ES cell pluripotentcy markers in shRNAi pools or parental ES cells by sqRT-PCR. For (B) comparison of all matched time points post-RA-treatment between control and *Ubr5* shRNAi pools revealed statistically significant differences (p = <0.01), apart from the comparison between control and *Ubr5*.*2* at 24 hours, which was not significant (ns). Statistical analysis by one-way ANOVA and Tukey multiple comparison test.

Due to the reciprocal association of UBR5 and *Shh* expression levels, we reasoned that, similar to Hyd’s ability to suppress *hh* expression [15, 16], UBR5 also functioned as a negative regulator of RA-mediated *Shh* expression. To address this we created stable pools of mES cells lines expressing three different *Ubr5* shRNAis or a control *scrambled* (*SCR*) shRNAi. Western blot analysis revealed normal levels of UBR5 expression in the SCR pool, but moderate (*Ubr5*.*1*), intermediate (*Ubr5*.*3*) and strong (*Ubr5*.*2*) reductions in UBR5 expression in *Ubr5* shRNAi pools (Fig 2A). We next challenged the *SCR* and *Ubr5* shRNAi pools (*Ubr5*.*1* and *Ubr5*.*2*) with RA and assessed their ability to express *Shh by* qRT-PCR (Fig 2B). In comparison to the *SCR* control line, both *Ubr5* shRNAi pools exhibited dramatically impaired *Shh* expression responses to RA stimulation. Interestingly, the initial *Shh* induction at 24 hrs was either unaffected (*Ubr 5*.*2*) or enhanced (*Ubr5*.*1*) over *SCR* control levels. However, both *Ubr5* shRNAi pools failed to significantly increase *Shh* expression over time. In comparison to the maximal *Shh* expression achieved in the SCR pool at 72hrs post RA-treatment, *Ubr5* shRNAi pools exhibited an approximately three-to-five-fold decrease. We therefore concluded that in our ES cell system UBR5 promoted RA-mediated *Shh* expression. This finding was in contrast to the identification of Hyd as a suppressor of *hh* expression [15], but did support a potential role for UBR5 in regulation of *hh* ligand expression.

The inability of *Ubr5* shRNAi cells to maintain *Shh* expression could have been explained by (i) an inability of ES cells to produce *Shh* or (ii) differentiation of ES cells into a non-*Shh*-expressing cell types. To address the latter, we assessed expression of a number of ES-cell associated markers (*Nanog*)[42], *Klf-4*[43] and *Bmp-4*[42, 44]. sqRT-PCR analysis revealed no dramatic differences in expression levels between *SCR* and *Ubr5*shRNAi pools (Fig 2C). Therefore, based on expression analysis of a limited set of markers, *Ubr5* shRNAi did not appear to affect ES pluripotentcy. While we cannot rule out a contribution of altered ES differentiation, we believe UBR5 cells plays a more direct role in influencing RA-mediated *Shh* expression within ES cells.

### *Ubr5*^*mt/mt*^ embryos phenocopy the *Ubr5* null phenotype

**Fig 3:**
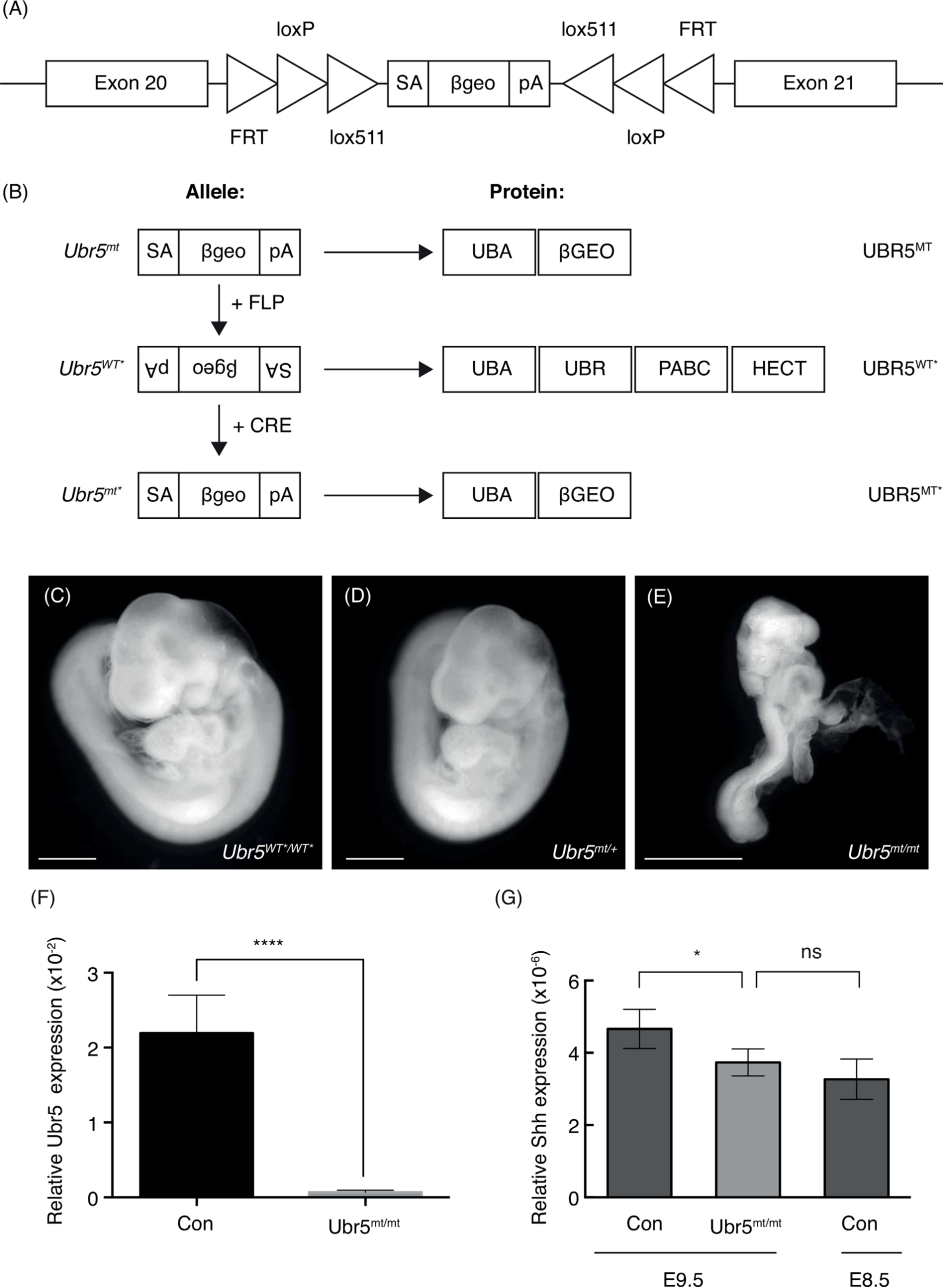
The *Ubr5* gene trap phenocopies the *Ubr5* null phenotype. (A) Schematic representation of the EUCOMM EUCE0171f01 gene trap introduced in between exons 20 and 21 of the murine *Ubr5* gene. *FRT*, *loxP* and *lox511* recombination sites, and their orientation, are indicated as triangles and flank the gene trap encoding for a splice acceptor site (SA), a *LacZ* and *neomycin* CDS (*βgeo*) and a poly-adenylation signal (*pA*). (B) Schematic representation of the effects of FLP and Cre-mediated recombination on the gene trap and UBR5 protein/domain expression–see text for details. (C-E) Brightfield images of control (C) *Ubr5*^WT*/WT*^, (D) *Ubr5*^*mt/+*^ and (E) *Ubr5*^*mt/mt*^ E9.5 embryos. Scale bar = 1mm. Whole embryo qRT-PCR analysis of (F) *Ubr5* at E9.5 and (G) *Shh* expression in the indicated genotypes at E9.5 or E8.5. Con = *Ubr5*^*+/+*^. n = ≥ 6, s.e.m indicated. Statistical analysis by Students t-test. ns = not significant.

Due to its well-described roles in limb patterning and development[1], we chose to examine the effects of loss of *Ubr5* function in limb bud mesenchyme. As *Ubr5* null embryos are embryonic lethal [45] we developed a conditional mutant allele. We utilised a *EUCOMM Ubr5* conditional gene trap (*Ubr5*^*gt*^) inserted between exons 20–21 (Fig 3A) to interfere with *Ubr5* mRNA expression and UBR5 protein function. The gene trap was capable of existing in three distinct states [46] depending on the sequential action of FLP- and CRE-recombinases (Fig 3B): (1) in the absence of recombinase the gene trap resided in the mutagenic orientation (*Ubr5*^*mt*^); (2) after FLP-mediated recombination, in the non-mutagenic orientation (*Ubr5*^WT*^); and (3) after CRE-mediated recombination, in the mutagenic orientation (*Ubr5*^mt*^). When in the mutagenic orientation the predicted fusion protein consists of UBR5’s N-terminal UBA domain fused with a β-galactasidase:aminoglycoside 3' phosphotransferase fusion protein (βGEO); with the βGEO protein conferring both X-gal staining and Neomycin resistance. Such a fusion of UBR5’s UBA domain with βGEO, herein referred to as UBR5^MT^, was predicted to be severely functionally impaired due to lack of its important domain-associated functions: (i) E3 activity due to loss of the C-terminal catalytic HECT domain [20], (ii) N-end rule function through loss of the UBR domain [17] and (iii) miRNA regulatory function due to the absence of its Poly(A)-binding protein C-terminal (PABC) domain [47].

To confirm that the UBR5^MT^ fusion protein was functionally impaired, we compared FLP-treated (*Ubr5*^WT*/WT*^) controls (Fig 3C), heterozygous (*Ubr5*^*mt/+*^) (Fig 3D) and homozygous (*Ubr5*^*mt/mt*^*) Ubr5*^*mt*^ (Fig 3E) E9.5 embryos. *Ubr5*^*mt/mt*^ embryos, in comparison to control or heterozygous embryos, appeared developmentally abnormal (Fig 3E), with an estimate of the apparent development stage being around E8.5. As expected these animals also exhibited a dramatic decrease in *Ubr5* expression (Fig 3F). Furthermore, qRT-PCR analysis of whole embryo extracts revealed a small, but significant, decrease in *Shh* expression in E9.5 *Ubr5*^*mt/mt*^ embryos (Fig 3G). This observation alone provided some support to the idea that UBR5 influenced *Shh* expression in vivo, even though the effects were small. However, we reasoned that the reduction in *Shh* expression might have simply reflected the developmental retardation of *Ubr5*^*mt/mt*^ embryos. In agreement, *Ubr5*^*mt/mt*^ *Shh* expression levels more closely resembled that of control E8.5 embryos (Fig 3G). These observations suggested that *Ubr5*^*mt/mt*^ embryos exhibited molecular signatures (i.e., reduced *Shh* expression) that potentially reflected their state of embryonic retardation.

To ensure that the *Ubr5*^*mt*^ allele functionally phenocopied the embryonic lethality of the null allele [45], we analysed the progeny of a heterozygous cross (*Ubr5*^*mt/+*^). The resultant litters revealed a total absence of *Ubr5*^*mt/mt*^ animals (Table 1). Hence, we were reassured that the *Ubr5* gene-trap functionally phenocopied the *Ubr5* null.

**Table 1:** E11.5 *Ubr5*^mt/mt^ embryos are not viable.

Genotype	Expected Frequency (%)	Observed Frequency (%)
**Ubr5^+/+^**	25%	26% (n = 7)
**Ubr5^mt/+^**	50%	74% (n = 20)
**Ubr5^mt/mt^**	25%	0% (n = 0)

Embryos from a *Ubr5*^*mt/+*^ cross were genotyped and their representation expressed as a percentage of the total litter size. n = 27. Chi squared test p = 0.0071.

### *Ubr5*^*mt/mt*^ embryos do not resemble *Shh* null embryos

**Fig 4:**
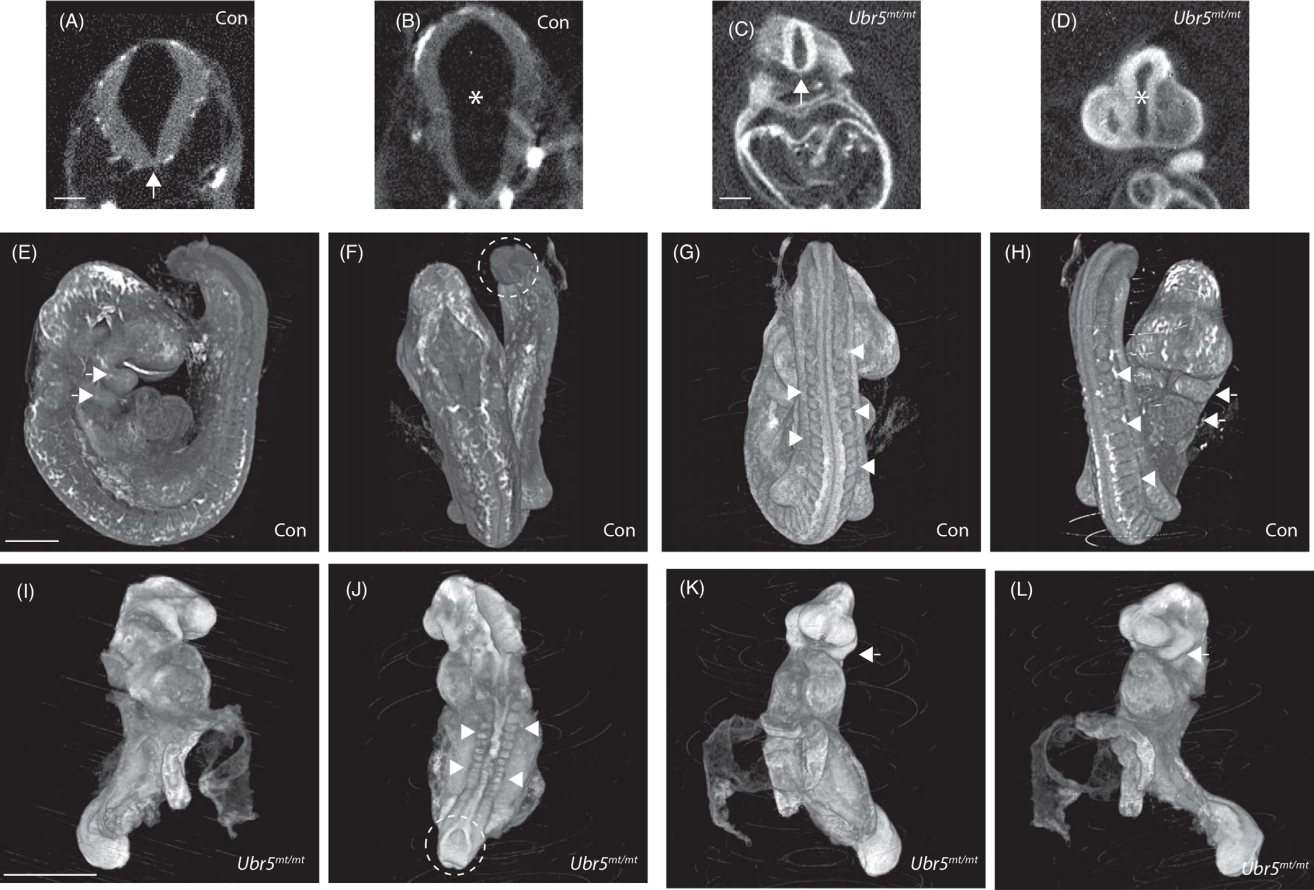
E9.5 *Ubr5*^mt/mt^ embryos are developmentally abnormal. Optical projection tomography images of *Ubr5*^*+/+*^ (Con) (A,B, E-H) and *Ubr5*^*mt/mt*^ (C,D, I-L) E9.5 embryos. Both *Ubr5*^*mt/mt*^ and Con embryos formed a neural floorplate (A,C arrows) and separated the plates of the diencephalic-mesencephalic junction (B,D asterisks). In comparison to control embryos, E9.5 *Ubr5*^*mt/mt*^ embryos exhibited numerous developmental defects: an open posterior neuropore (J dashed line), irregular somites that were also reduced in number (compare G,H with J, arrowheads); lordotic curvature (L) and only one pair of pharyngeal arches (compare E,H with K,L arrows). n = 1. Scale bar (A-C) = 200μm and (E-L) = 1mm.

We next used optical projection tomography (OPT) to compare control (*CD1*) and *Ubr5*^*mt/mt*^ E9.5 embryos in more detail (Fig 4). Specifically we wished to address whether the *Ubr5*^*mt/mt*^ embryos bore any resemblance to *Shh* defective embryos. Embryos deficient in *Shh* die during late embryonic development and display disruption of midline structures, cyclopia and limb deformities >E10.5 [48, 49]. Unfortunately, the death and reabsorption of *Ubr5*^*mt/mt*^ embryos by E11.5 precluded detection of any *Shh*-associated mid-gestation phenotypes. However, additional *Shh* null defects that present earlier than E10.5 were not apparent in E9.5 *Ubr5*^*mt/mt*^ embryos: namely defects in the neural tube / floor plate (Fig 4 compare A and C, arrows) or closure of the dorsal and ventral surfaces around the dicephalic-mesencephalic junction (Fig 4 compare B and D, asterisk). Nevertheless, OPT did confirm that the morphology of these structures were clearly different to that of the age matched control embryos.

Further comparisons revealed that the E9.5 *Ubr5*^*mt/mt*^ animals exhibited (i) kyphotic, rather than lordotic, curvature of the spine (Fig 4, compare E,I), (ii) an open, rather than closed, posterior neuropore (Fig 4, compare F,J dashed lines), (iii) one, rather than two, clearly defined pharyngeal arches (Fig 4, compare E,H with K,L, arrows) and (iv) ~12, rather than 24, somite pairs (Fig 4, compare G,H with J, arrowheads). Furthermore, there was some evidence of somite misalignment (Fig 4J), a defect associated with loss of *Shh* function [49].

Taken together these results supported the idea that *Ubr5*^*mt/mt*^ embryos were clearly developmentally abnormal. However the E9.5 *Ubr5*^*mt/mt*^ embryos did not resemble *Shh* null animals, but did phenocopy the previously reported *Ubr5* null morphological phenotype [45].

### UBR5 function is required for mid-gestation embryonic viability

To address the role of *Ubr5* at later stages and to examine any Hh-associated defects occurring during midgestation (>E10.5) we coupled the conditional *Ubr5*^WT*^ allele with the tamoxifen inducible *Cre-ER*^*T2*^ system [50] driven by expression from the artificial *CAGG* promoter [51]. Pregnant females bearing *Cre-ER*^*T2*^*;Ubr5*^WT*/WT*^ progeny were injected daily with tamoxifen at E10.5 over four days. Genotyping the embryos at E15.5 revealed a total absence of the *Cre-ERT*^*2*^*; Ubr5*^mt*/mt*^ genotype and a reduction in the observed/expected frequency of *Cre-ERT*^*2*^*; Ubr5*^mt*/+^ embryos (Table 2). In a further attempt to recover *Ubr5*^mt*/mt*^ embryos for morphological analysis, we repeated the experiment and harvested the embryos at E13.5. Again, E13.5 analysis revealed the same absence of the *Cre-ERT*^*2*^*; Ubr5*^mt*/mt*^ embryos (Table 3), as well as a detrimental effect on heterozygous embryo viability (Tables 2 and 3). These results suggested that during midgestation *Ubr5* gene dosage was important for its embryonic function. Importantly, tamoxifen-mediated Cre activity alone (*Cre-ER*^*T2*^) had no detrimental effects on embryonic viability. Taken together this data suggested that the *Ubr5*^WT*^ allele was amenable to Cre-mediated conversion into the *Ubr5*^mt*^ allele and that UBR5 function was required for mid-gestation embryonic viability.

**Table 2:** *Ubr5* is required for embryonic mid-gestational viability—E15.5 analysis.

Genotype	Expected Frequency (%)	Observed Frequency (%)
**Ubr5^+/+^**	12.5	14 (n = 5)
**Ubr5^+/+^; CreER^T2^**	12.5	25 (n = 9)
Ubr5^WT*/+^	25	36 (n = 13)
Ubr5^mt*/+^; CreER^T2^	25	11 (n = 4)
Ubr5^WT*/WT*^	12.5	11 (n = 4)
Ubr5^mt*/mt*^; CreER^T2^	12.5	0 (n = 0)

**Table 3:** *Ubr5* is required for embryonic mid-gestational viability—E13.5 analysis.

Genotype	Expected Frequency (%)	Observed Frequency (%)
**Ubr5^+/+^**	12.5	20 (n = 7)
**Ubr5^+/+^; CreER^T2^**	12.5	29 (n = 10)
Ubr5^+/WT*^	25	20 (n = 7)
Ubr5^+/mt*^; CreER^T2^	25	3 (n = 1)
Ubr5^WT*/WT*^	12.5	29 (n = 10)
Ubr5^mt*/mt*^; CreER^T2^	12.5	0 (n = 0)

*Ubr5* function is required for mid-gestation viability. Pregnant females were injected i.p with tamoxifen at E11.5 and embryos genotyped at E13.5 (Table 3) and E15.5 (Table 2). Chi squared analysis: E15.5 = p 0.0179 and E13.5 = p = 0.0001.

### *Ubr5*/UBR5 is expressed in the limb buds

**Fig 5:**
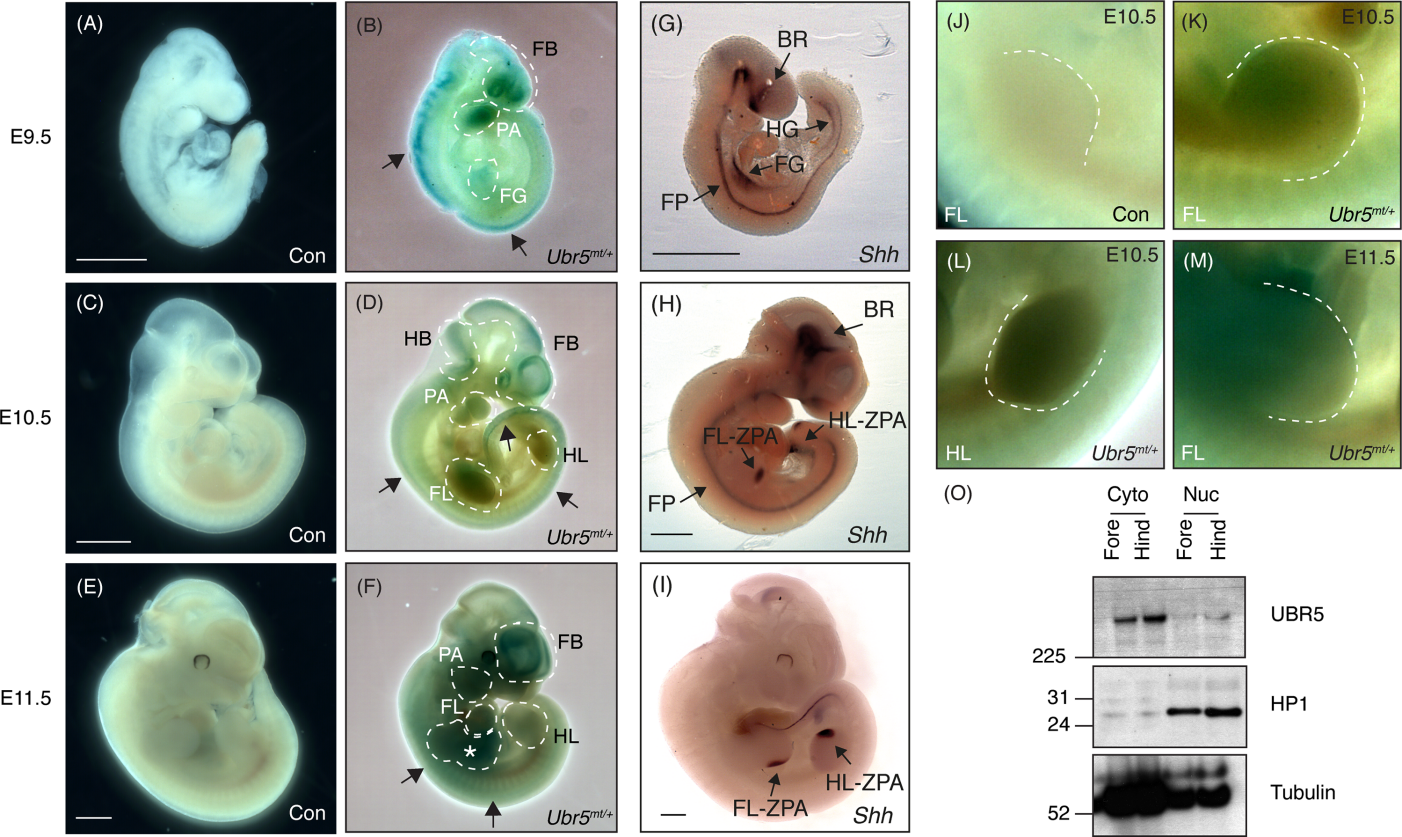
Expression analysis of UBR5^MT^, *Shh* and endogenous UBR5. β-Gal activity was not detected in wild-type controls (Con) embryos (A,C,E), whereas Ubr5^mt/+^ embryos exhibited staining at (B) E9.5, (D) E10.5 and E11.5 (F). Dashed lines indicate areas of interest. Please see text for more details. (G-I) *Shh* in situ hybridisation of wild-type control embryos at the indicated embryonic stages. FB = forebrain; HB = hindbrain; PA = pharyngeal arches; FG = foregut; HG = hindgut; FL = forelimb; HL = hindlimb; BR = brain; FP = floorplate; ZPA = zone of polarising activity; an asterisk marks a potential gastrointestinal signal. Scale bars = 1mm. (J-M) Higher magnification images of E10.5 (J-L) and E11.5 (M) limbs of the indicated genotypes. A dashed line outlines the limb bud margins. Representative images shown from n = ≥3 for each stage and genotype. (O) SDS-PAGE and Western blotting of E11.5 fore- and hind-limb lysates for UBR5 expression in cytosolic (Cyto) and nuclear (Nuc) fractions. HP1 (a nuclear marker) and β-tubulin (a predominantly cytosolic marker) were used as loading and fractionation controls. n = 1.

Once the *Ubr5*^*gt*^ construct had been functionally validated, we chose to utilise the gene trap’s *lacZ* reporter gene to establish UBR5 protein expression, with a particular focus on the limbs. E9.5, E10.5 and E11.5 embryos heterozygous for *Ubr5*^*mt*^ underwent X-Gal staining (Fig 5A–5F). No significant staining was detected in control *Ubr5*^*+/+*^ embryos (Fig 5A, 5C and 5E). In contrast, *Ubr5*^*mt/+*^ embryos at E9.5, E10.5 and E11.5 (Fig 5B, 5D and 5F, respectively) exhibited widespread low-level X-Gal staining in addition to well-defined stronger signals. At all stages *Ubr5*^*mt/+*^ embryos revealed distinct signals associated with the dorsal edge (Fig 5B, 5D and 5F arrows) in addition to signals in the pharyngeal arches and forebrain (Fig 5B, 5D and 5F dashed lines labelled PA and FB, respectively). At E9.5, a signal within the body core potentially indicated expression within the foregut (Fig 5B, labelled FG). At E10.5, the staining along the dorso-lateral edge shifted posteriorly towards the tail (Fig 5D, posterior arrow) and by E11.5, the striated β-Gal activity was still present along the dorsal edge, but had become more medial (Fig 5F, arrows). Additionally, X-Gal staining became more prominent in the forebrain (Fig 5 dashed lines, FB) and body core (Fig 5F asterisk). Staining in both fore- and hind-limbs was detected at E10.5–11.5, with the fore-limbs exhibiting more robust staining (Fig 5D–5F dashed lines, FL and HL). In summary, the β-Gal activity exhibited dynamic expression patterns in a number of structures that included, at certain stages, both fore- and hind-limbs.

We next wished to compare UBR5-associated β-Gal activity with *Shh* expression patterns. *Shh* in situ hybridisation at E9.5, E10.5 and E11.5 (Fig 5G–5I) revealed the expected expression in the fore- and hind-gut (labelled FG and HG, respectively), brain (labelled BR), floorplate of the neural tube (labelled FP) and the ZPAs of the fore- and hind-limbs (labelled FL-ZPA and HL-ZPA, respectively). By E11.5 *Shh* expression was predominantly restricted to the limb buds and the zones of polarising activity (Fig 5H and 5I labelled ZPA). In contrast to the posterioirised *Shh* expression in the forelimb, UBR5-associated β-Gal activity appeared to be predominantly in the anterior regions of the E10.5 and E11.5 forelimb buds (Fig 5K–5M, respectively). However, in the E10.5 hindlimb β-Gal activity was evenly expressed across the entire limb bud (Fig 5L). In general, the overall expression patterns of *Shh* and UBR5-associated β-Gal activity revealed no strong evidence for either a clear mutually-exclusive or -inclusive expression pattern. However, *Ubr5* expression within the limb buds did provide a possibility for UBR5 to tissue autonomously regulate Hh signalling within the embryonic limb.

The β-Gal assays suggested that *Ubr5*/UBR5^mt^ was expressed in E10.5 and E11.5 limb buds. To confirm that endogenous UBR5 protein was also expressed, we used SDS-PAGE and Western blotting of E11.5 limb lysates (Fig 5O). Samples were separated into crude nuclear and cytoplasmic fractions and blotted for UBR5 and cytoplasmic (tubulin) and nuclear (HP-1) markers. Analysis revealed that UBR5 was expressed in both the fore- and hind-limbs and was present in both the cytoplasmic and nuclear fractions. Due to potential cytoplasmic contamination, as indicated by the presence of β-tubulin in the nuclear fraction, we were unable to conclude that UBR5 is nuclear localised in limb bud cells. Nevertheless, techniques to identify UBR5:βGeo-associated β-Gal activity (Fig 5B, 5D, 5F and 5K–5M) and endogenous protein expression (Fig 5O) revealed that *Ubr5*/UBR5 was expressed in E11.5 embryonic limb buds.

### *Prx-Cre1*-mediated recombination of the *Ubr5*^WT*^ allele effectively suppresses *Ubr5* expression in developing limbs

**Fig 6:**
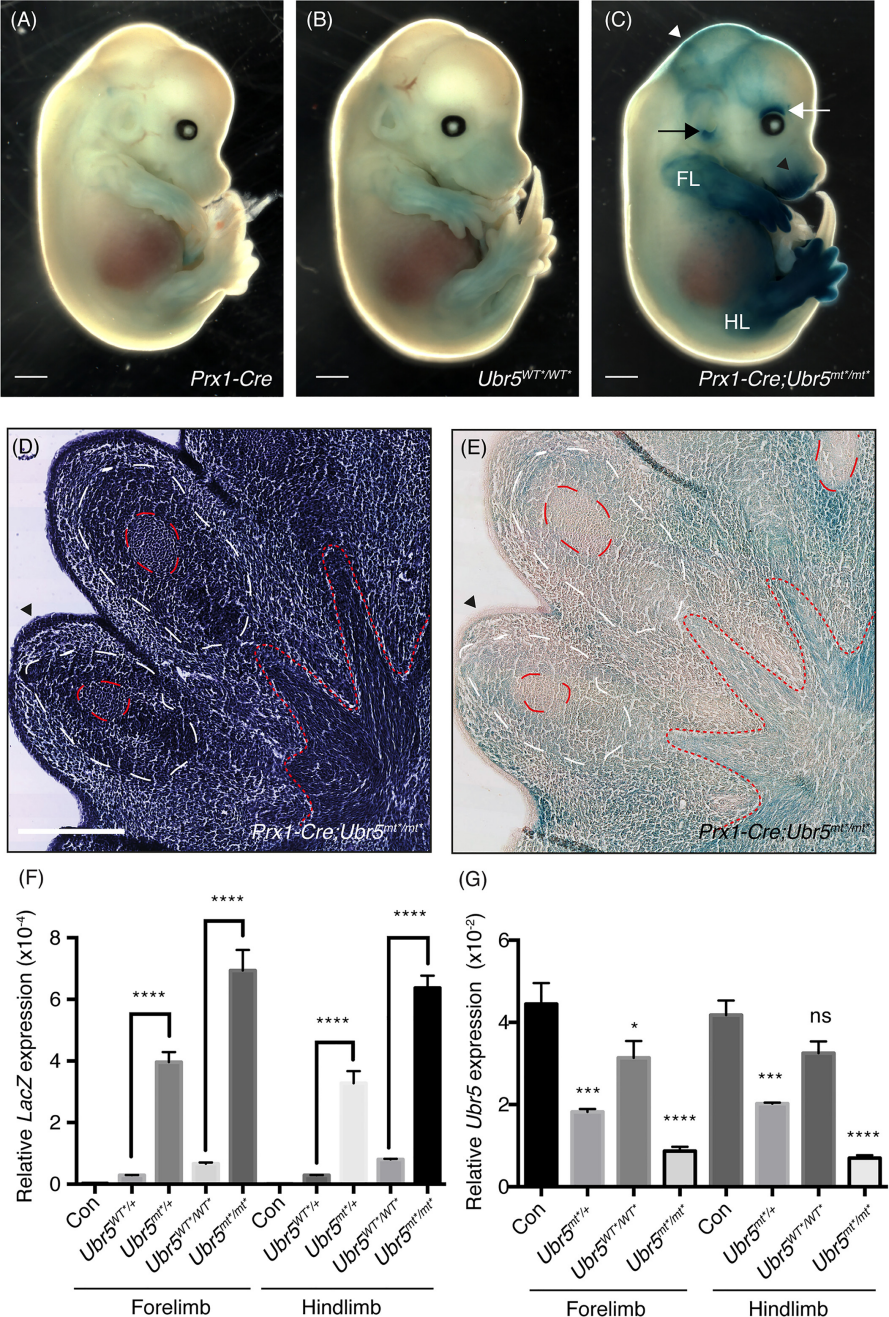
*Prx-Cre1*-mediated recombination of the *Ubr5*^gt^ and loss of *Ubr5* expression. *Prx1*-Cre promotes UBR5^MT^-associated β-Gal activity (A-E), lacZ expression (F) and (G) loss of *Ubr5* expression. (A-C) E13.5 embryos of the indicated genotypes stained for β-Gal activity revealed strong X-Gal staining in the embryonic limbs, in addition to other regions: above the eye, white arrow; the ear, black arrow; snout black arrowhead and head, white arrowhead. Scale bar = 1mm. (D-E) Histological sections through the footplate revealed a non-uniform UBR5^MT^ expression pattern (red dashed lines = chondrocyte condensations; white dashed lines = dark staining mesenchyme and red dotted line = developing sinews). Scale bar = 250μm. qRT-PCR analysis of (F) *LacZ* expression and (G) full-length *Ubr5* in dissected E13.5 fore- and hind-limb buds. *Prx1-Cre* in combination with heterozygosity or homozygosity for the *Ubr5*^mt*^ allele exhibited (F) a dramatic increase in *LacZ* expression and (G) a dramatic decrease in *Ubr5* expression in comparison to *Prx1-Cre* only (Con). The primer pairs in (G) were specific for a gene region absent in the *Ubr5*^mt*^ transcript. All differences relative to the appropriate *Prx1-Cre* controls (Con) were statistically significant (at least p = <0.05), apart from the comparison between Con and *Ubr5*^mt*/mt*^, which was not significant (ns). n = ≥3, s.e.m indicated. Statistical analysis by one-way ANOVA and Tukey multiple comparison test.

The combination of (i) our observation of UBR5’s effects on RA-mediated *Shh* expression, (ii) the importance of RA and SHH-mediated signalling in limb development and (iii) *Ubr5*/UBR5 expression within the limb prompted us to delete UBR5 expression in the developing limb. Accordingly, we chose to combine the *Ubr5*^WT*^ allele with *Prx1*-mediated expression of *Cre* recombinase (*Prx1-Cre*). Expressed throughout the E9 limb bud mesenchyme, *Prx-1*-Cre mediates recombination in all mesenchymal cells by mid-bud development at E11 [36]. To confirm the efficiency of *Prx1*-Cre-mediated recombination, control and experimental E13.5 embryos were assayed for β-gal activity (Fig 6A–6C). Note that F1 progeny bearing both *Prx1-Cre* and *Ubr5*^WT*/WT*^ were assumed to have undergone temporally- and spatially-restricted recombination and from herein will be indicated as *Prx1-Cre;Ubr5*^mt*/mt*^.

*Prx-Cre;Ubr5*^mt*/mt*^ embryos exhibited robust β-gal activity within the limbs (Fig 6C) that was absent in *Ubr5*^*+/+*^ and *Ubr5*^WT*/WT*^ controls (Fig 6A and 6B, respectively). In *Prx-Cre;Ubr5*^mt*/mt*^ embryos, significant signals were also detected outside of the limbs and included regions above the eye (Fig 6C white arrow), around the ear (Fig 6C black arrow), the snout (Fig 6C black arrowhead) and head (Fig 6C, white arrowhead). These observations indicated that both *Prx1-Cre* and *Ubr5* were expressed within, as we well as outside of, the limb fields. In summary, these results confirmed (i) efficient Cre-mediated gene conversion and (ii) *Ubr5*/UBR5 expression within the fore- and hind-limbs.

Haematoxylin-based histological examination of the *Prx-Cre;Ubr5*^mt*/mt*^ footplate revealed a dark staining epidermal layer (Fig 6D, arrowhead), chondrocyte condensations within the developing digits and surrounding dark staining mesenchymal cells (Fig 6D, red and white dashed lines respectively). Proximally extending structures indicative of developing sinews radiated out from the mid-region of the footplate (Fig 6D, red dotted lines). Examination of an adjacent unstained section revealed widespread β-Gal activity (UBR5^MT^) across the footplate (Fig 6E). Regions of high expression appeared to reside around and between the chondrocyte condensations as well as within the developing sinews. In contrast, β-Gal activity appeared to be either low or absent within the chondrocyte condensations and epidermis (Fig 6, red dashed line and arrowhead, respectively). In summary, within the *Prx1-Cre;Ubr5*^mt*/mt*^ forelimb, UBR5^MT^ exhibited a non-uniform expression pattern and appeared to be excluded from condensing chondrocytes.

We next carried out qRT-PCR analysis on *Prx-Cre;Ubr5*^mt*/mt*^ limbs to quantify the extent of gene conversion through increased *LacZ* expression (Fig 6F) and confirm that *Ubr5* expression was also reduced (Fig 6G). *LacZ* primers targeting expression of *Ubr5*^mt*^ mRNA revealed no signal in *Prx1-Cre* animals, but detected low-levels in heterozygous and homozygous *Ubr5*^WT*^-bearing animals that lacked *Prx1-Cre*. Nevertheless, the presence of *Prx1-Cre* promoted a 15–40 fold increase in *LacZ* expression in either heterozygous or homozygous *Ubr5*^mt^* backgrounds (Fig 6F). Interestingly *Prx1-Cre*;*Ubr5*^mt*/mt*^ exhibited only ~33% more *LacZ* expression than *Prx1-Cre;Ubr5*^mt*/+^, suggesting either non-linear gene dosage effects and/or limiting recombinase activity.

To quantify *Ubr5* expression, we used primer pairs complementary to the 3’ end of *Ubr5* mRNA to discriminate endogenous and *Ubr5*^WT*^ from *Ubr5*^mt*^, alleles. In agreement with the increased β-Gal staining (Fig 6C) and *LacZ* expression analysis (Fig 6F), full-length *Ubr5* expression levels were significantly reduced in *Prx1-Cre* limbs either heterozygous or homozygous for *Ubr5*^mt*^ (Fig 6G); with the greatest reduction occurring in the homozygous *Ubr5*^mt*/mt*^ limbs. Surprisingly, homozygosity for the non-mutagenic *Ubr5*^WT*^ allele, in the absence of *Prx1-Cre*, caused a small but significant decrease in *Ubr5* expression. This observation indicated that physical insertion of the gene-trap (Fig 6F) had a detrimental effect on *Ubr5* mRNA expression.

### *Ubr5*^mt^ E13.5 limbs exhibit decreased Hedgehog pathway activity

**Fig 7:**
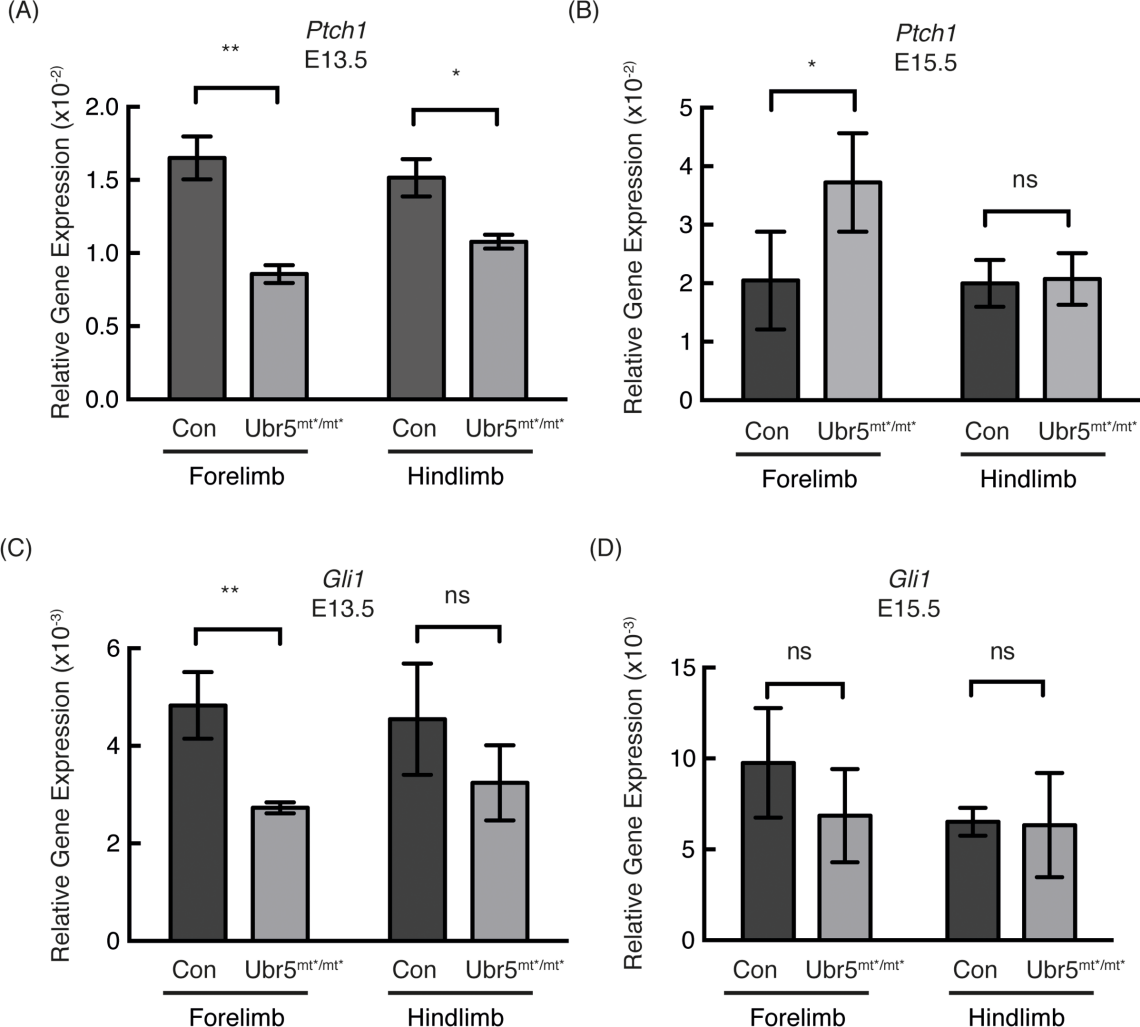
Loss of *Ubr5* function resulted in decreased expression of *Ptch1* and *Gli1* at E13.5. (A-D) qRT-PCR analysis of *Prx1-Cre* (Con) and *Prx1-Cre;Ubr5*^mt*/mt*^ (*Ubr5*^mt*/mt*^) E13.5 or E15.5 embryonic fore- and hind-limbs for *Ptch1* (A,B) and *Gli1* (C,D) expression. qRT-PCR of target genes were normalised against β-actin (n = ≥3, s.e.m indicated). Statistical analysis by Students t-test. ns = not significant.

Having established that *Ubr5* was expressed in the limb and that the *Ubr5*^*gt*^ was functional, we wanted to address whether loss of *Ubr5* expression in the developing limb would affect Hedgehog signalling (Fig 7). qRT-PCR analysis of the Hh pathway target gene *Ptch1* revealed that at E13.5 HhP activity was repressed in both the fore- and hind-limb (Fig 7A). At E15.5, the levels of *Ptch1* expression appeared to be either overcorrected (forelimb) or corrected (hindlimb) relative to control levels (Fig 7B). Similar to *Ptch1* expression, *Gli1* expression was also repressed in both limbs, although only the reduction in the forelimb was statistically significant (Fig 7C) and was then corrected, to some degree, by E15.5 (Fig 7D).

In summary, at E13.5 it appeared that loss of *Ubr5* in the forelimb resulted in a decrease in the HhP’s transcriptional outputs. Within the hindlimb, however, the reduction in HhP transcriptional output was less pronounced, Some of the differences between fore- and hind-limbs may have reflected the difference in developmental timing between the two structures, with the forelimb initiating development prior to the hindlimb (EMouse Atlas Project). Overall, these results suggested that loss of *Ubr5* function resulted in a transient repression of Hh signalling.

**Fig 8:**
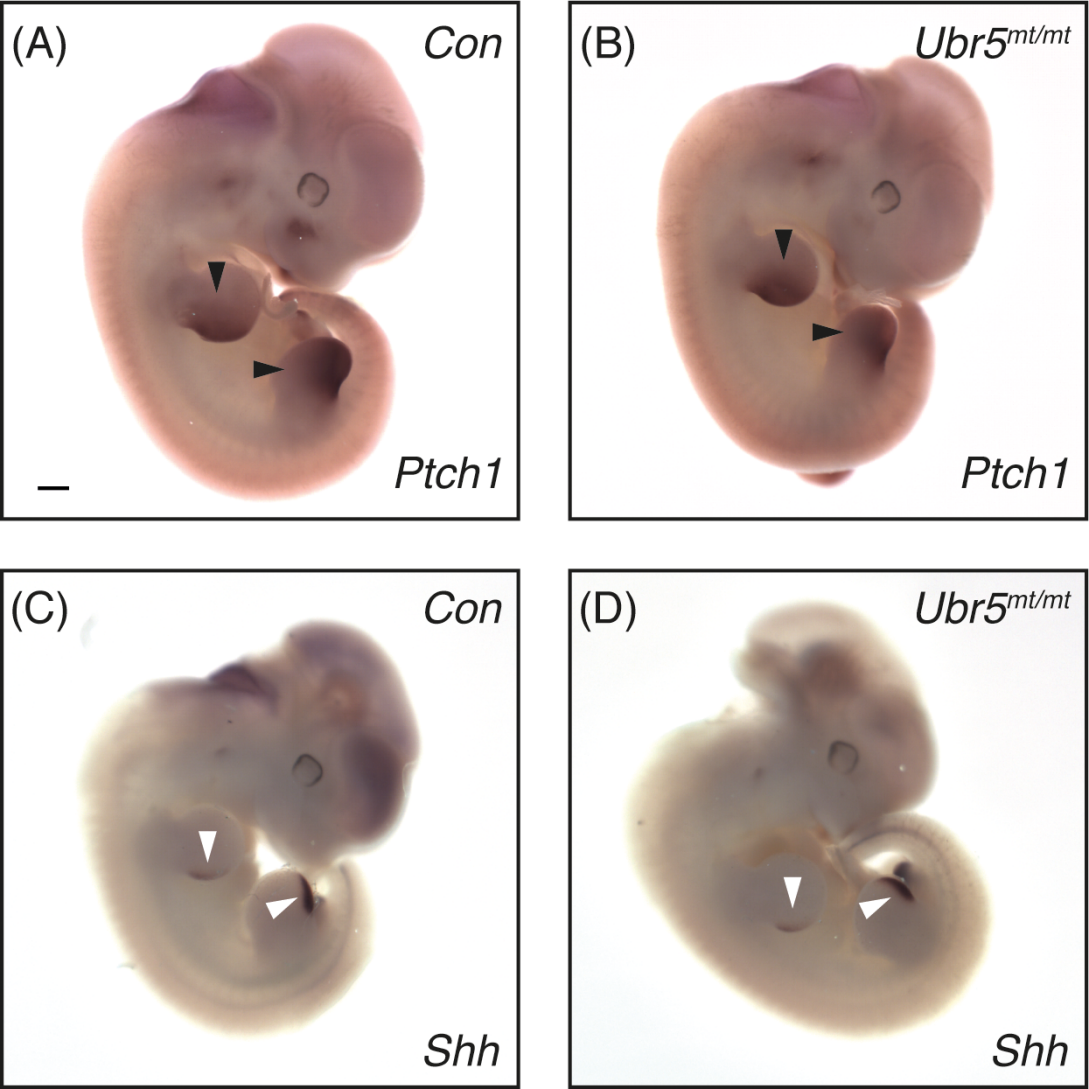
Loss of *Ubr5* at E11.5 does not affect *Shh* or *Ptch1* expression domains. In situ hybridisation for expression of Hh signalling components *Ptch1* (A,B) and *Shh* (C,D) in E11.5 embryos of *Prx1-Cre* (Con) and *Prx1-Cre;Ubr5*^mt*/mt*^ (*Ubr5*^mt*/mt*^) embryos. Analysis revealed no significant effects on either *Shh* or *Ptch1* expression patterns in the *Ubr5*^mt*/mt*^ embryos (representative image from n = >4 of each genotype). Arrowheads indicate *Shh* ZPA expression domains. Scale bar = 0.5mm.

Significant quantitative changes in *Ptch1* gene expression at E13.5 led us to investigate its spatial expression pattern at E11.5. Using ISH, we examined *Ptch1* expression in both fore- and hind-limb buds of E11.5 embryonic fore- and hind-limbs of control *Prx1-Cre* (Fig 8A) and experimental *Prx1-Cre; Ubr5*^*mt/mt*^ (Fig 8B) embryos. Comparison between the two genotypes revealed no dramatic differences in spatial expression patterns, although Ubr5^mt/mt^ hindlimbs exhibited a small reduction in *Ptch1* signal intensity (Fig 8B). Analysis of *Shh* expression showed no differences in its spatial expression patterns (Fig 8C and 8D). Taken together these data revealed *that Prx1-Cre*-mediated loss of UBR5 function did not dramatically affect *Shh* or *Ptch1* spatial expression patterns in E11.5 embryonic limbs. In combination with the qRT-PCR analysis, we concluded that in *Ubr5mt* limbs the effect on Hedgehog signalling were transient and predominantly affected the magnitude of mRNA expression around E11.5-E13.5.

### *Ubr5*-deficient embryonic limbs do not exhibit digit abnormalities

**Fig 9:**
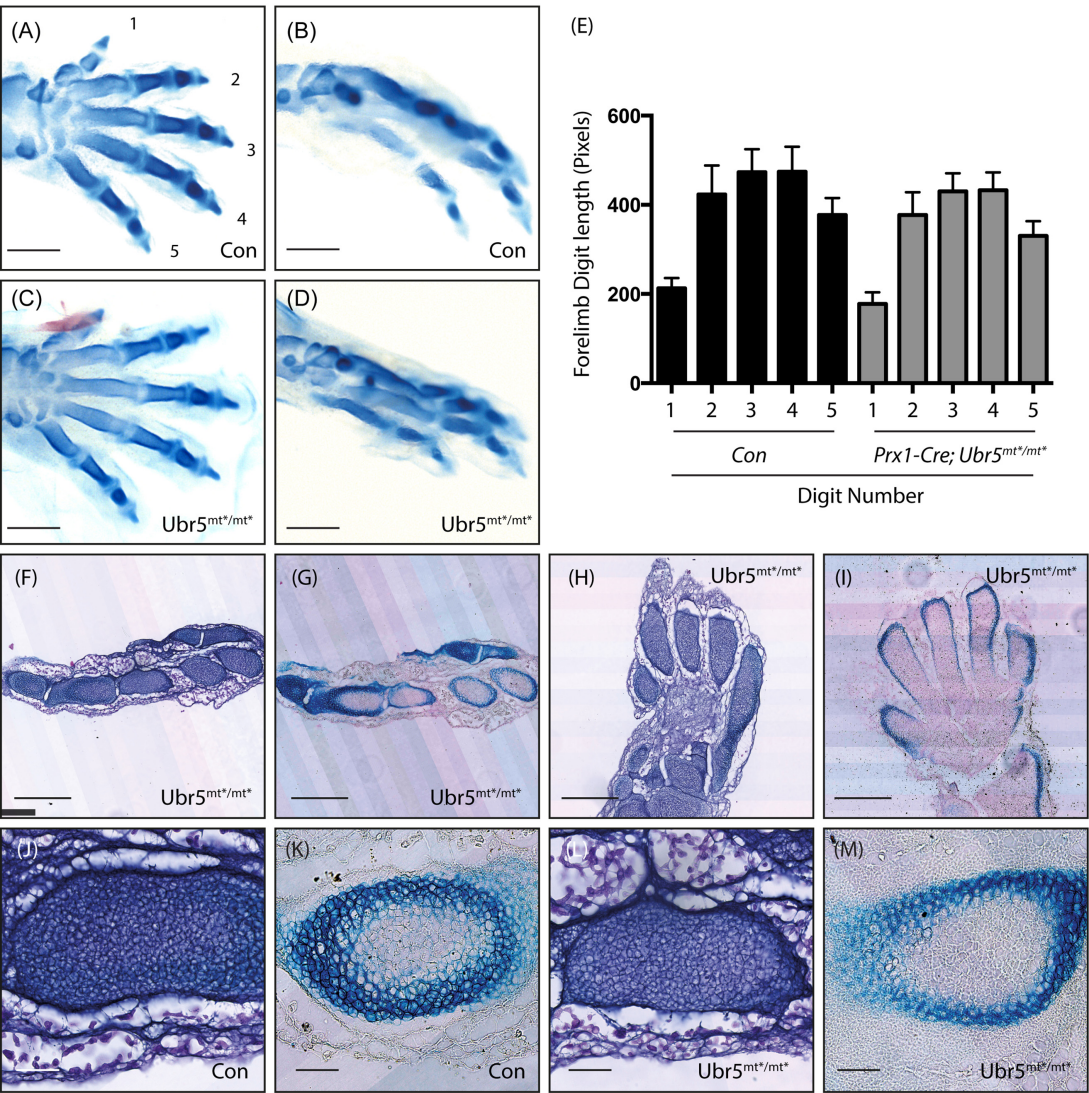
*Ubr5*^mt*/mt*^ limbs and digits appear morphologically normal. Analysis of fore- (A,C, H, I) or hind-paws (B,D,F,G, J-M) of *Prx1-Cre* (Con) and *Prx1-Cre;Ubr5*^mt*/mt*^ (*Ubr5*^mt*/mt*^) E15.5 embryos. (A-D) No obvious morphological difference were apparent between Con (A,B) and *Ubr5*^mt*/mt*^ (C,D) digits. Scale bar = 1mm. (E) Measurement of forelimb digit length, numbered as in (A), revealed a reduction in the average length in *Ubr5*^mt*/mt*^ animals. However, none of the comparisons between matching digits were statistically significant (p = >0.05). n = ≥6, s.e.m indicated. Statistical analysis by one-way ANOVA and Tukey multiple comparison tests. (F-M) Histological analysis of haematoxylin (F,H,J,L) and alcian blue (G,I,K,M) stained material. Histology and morphology of *Ubr5*^mt*/mt*^ hind- (F,G) or fore-paws (H,I) appeared histologically and morphologically normal, (J-M) as did chondrocyte clusters within the hind-paw. Scale bar = 250μm (F-I) and 50μM (J-M).

Although the E11.5 limb buds showed no obvious changes in the spatial expression of *Shh*, the E13.5 limbs did exhibit a reduction in HhP activity. We hypothesised that even a transient reduction might manifest a subsequent morphological/developmental defect. Accordingly, we examined the digits of E15.5 control *Prx1-Cre* and *Prx1-Cre*, *Ubr5*^mt*/mt*^ fore- and hind-paws, which revealed no gross abnormalities (Fig 9A–9D). Although measurement of *Ubr5*^mt*/mt*^ forelimb digits revealed a reduction in average length (Fig 9E), the effects proved not to be statistically significant.

Histological analysis of *Prx1-Cre;Ubr5*^mt*/mt*^ mutant feet and paws using haematoxylin or alizarin red/alcian blue revealed no obvious defects in digit formation, or composition (Fig 9F–9I). Closer analysis of *Prx1-Cre* and *Prx1-Cre;Ubr5*^mt*/mt*^ developing tarsals (Fig 9J–9M) also revealed no dramatic effects on chondrocyte condensations (Fig 9 compare J,L) or upon the associated deposition of Alcian-blue-reactive acidic polysaccharides (cartilage) (Fig 7 compare K,M). Therefore, it appeared that there were no obvious morphological/histological defects in *Ubr5*^mt*/mt*^ digits.

### *Ubr5*-deficient embryonic limbs exhibit reduced *Ihh* expression

**Fig 10:**
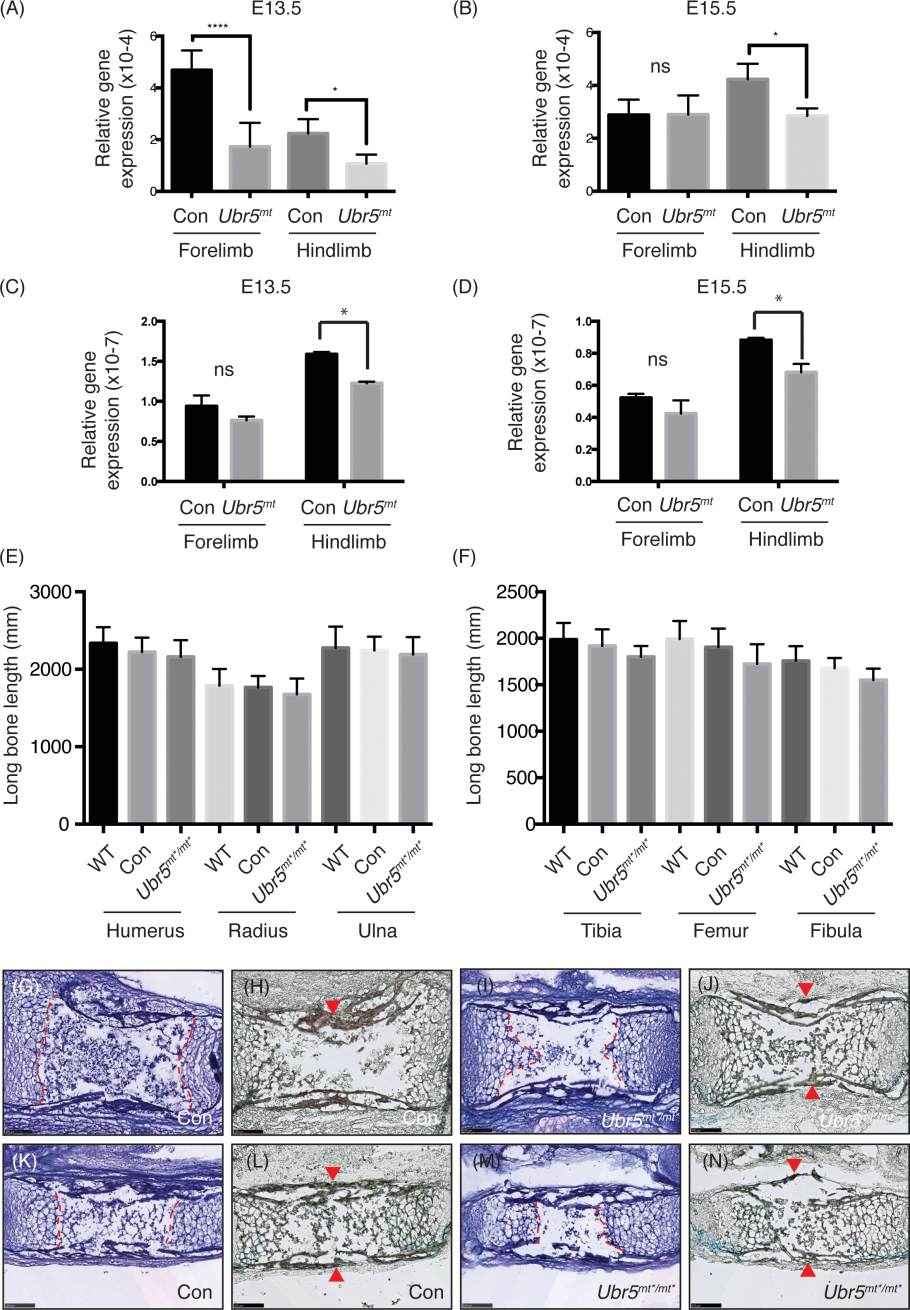
*Ubr5*^mt*/mt*^ limbs exhibit reduced *Ihh* expression. (A-B) *Ihh* and (C,D) *Shh* qRT-PCR analysis of control *Prx1-Cre* (Con) or *Prx1-Cre; Ubr5*^mt*/mt*^ (*Ubr5*^*mt*^) fore- or hind-limbs at (A,C) E13.5 or E15.5 (B,D) revealed significant decreases in *Ubr5*^*mt*^ limbs versus Con. qRT-PCR values were normalised against β-actin (n = 3, s.e.m indicated). Statistical analysis by Students t-test. (E,F) Measurement of the length of the indicated long bones in *Ubr5*^*+/+*^ (WT), *Prx1-Cre* (Con) or *Prx1-Cre;Ubr5*^mt*/mt*^ (*Ubr5*^mt*/mt*^) animals revealed no statistically significant reductions in length. Statistical analysis by ANOVA and Tukey multiple comparison test. (G-N) Histological analysis of haematoxylin (G,I,K,M) and alcian blue + alizarin red (H,J,L,N) stained humerus (G-J) and ulna (K-N) revealed no obvious histological or morphological differences. Dashed lines = border between chondrocytes and site of primary ossification and arrowheads = alizarin red positive cortical bone. Scale bar = 100μm.

The lack of any digit phenotype suggested that the observed changes in HhP activity had no impact on digit development. In simplistic terms, Shh-mediated signalling controls limb/digit formation; whereas, *Ihh* regulates the subsequent growth, maturation and homeostasis of the limbs’ long bones [52, 53]. We therefore hypothesised that reduced *Ihh* expression could account for the decrease in HhP activity and might elicit an effect on embryonic long bone length. qRT-PCR analysis revealed a significant reduction in *Ihh* expression in E13.5 *Prx1-Cre;Ubr5*^mt*/mt*^ limbs (Fig 10A), with only the reduction in the hind limbs persisting through to E15.5 (Fig 10B).

At E13.5-E15.5 *Shh* expression in the limb bud is dramatically reduced[54–56] and *Shh* analysis revealed no significant difference between control and *Prx1-Cre;Ubr5*^mt*/mt*^ fore limbs at either time point (Fig 10C and 10D). However, at both developmental stages, *Prx1-Cre;Ubr5*^mt*/mt*^ hindlimbs exhibited small, but significant decreases (Fig 10C and 10D). It should be noted that the *Shh* detected at >E13.5 was most likely in non-mesenchymal-derived developing hair follicles[57] and therefore not directly affected by *Prx1*-*Cre*-mediated loss of *Ubr5* function. Overall, our data suggested that a reduction in *Ihh*, rather than *Shh*, expression potentially accounted for the observed reduction in HhP activity at E13.5 (see Fig 7).

Due to IHH’s well-established role in regulating bone growth [52, 53] we measured the length of the *Prx1-Cre;Ubr5*^mt*/mt*^ long bones at E15.5 (Fig 10E and 10F). Similarly to what was observed in the digits, *Prx1-Cre;Ubr5*^mt*/mt*^ long bones were on average shorter than those of *Ubr5*^*+/+*^ (WT) or *Prx1-Cre* (Con) control animals. However, the effects were not statistically significant. Finally, we chose to examine the E15.5 long bones for morphological defects. As in the digits, histological analysis of the control and *Ubr5*^*mt*^ humerus (Fig 10E–10HJ) and ulna (Fig 10K–10N) revealed no obvious morphological differences or changes in alcian blue and alizarin red staining. In conclusion, loss of *Ubr5* function correlated with a transient decrease in *Ihh* expression, but no obvious or statistically significant limb defects.

## Discussion

In conclusion, our in vitro and in vivo findings support the initial hypothesis that UBR5 can regulate *hh* ligand family expression and HhP activity. However, we are uncertain as to how UBR5 influences Hedgehog signalling. One mechanism would involve an indirect route whereby UBR5 governs the production/maintenance of Hh-ligand producing / Hh-responsive cells. Such a mechanism could be extremely indirect, with defects occurring early on in development only manifesting a molecular or cellular consequence later on. Whereas a more direct role could involve UBR5 acting cell-autonomously to promote *hh* ligand mRNA expression and/or Hh pathway transcriptional outputs. Future efforts will attempt to resolve this uncertainty.

Our initial in vitro observations supported a cell autonomous role for UBR5 in promoting RA-mediated *Shh* expression. Although maximal *Shh* expression was impaired in UBR5-deficient ES cells, the initial RA-mediated induction was not. Therefore, these results potentially indicate two distinct phases in RA-mediated *Shh* expression: (i) an initial UBR5-independent induction phase and (ii) a subsequent UBR5-regulatable, amplification phase. However, we are uncertain as to how UBR5 may influence RA-mediated signalling. One possibility resides in UBR5’s ability to bind [58] the retinoic-acid responsive [59] progesterone receptor (PGR). Although no evidence exists that PGR induces *Shh* expression, it can promote *Ihh* expression [60, 61].

While our work supported a role for UBR5 in promoting *Shh* expression in ES cells, it was not supported by our in vivo studies. At E13.5 *Prx1-Cre;UBr5*^mt*/mt*^ hindlimbs exhibited only small decreases in *Shh* expression that were not apparent in the forelimbs. However, significant decreases in *Ihh* expression and HhP activity were detected in fore- and hind-limbs at E13.5. Therefore, in *Ubr5*^*mt*^ limbs reduced *Ihh* expression may have accounted for the reduction in HhP activity. The transient nature of these effects could indicate (i) a tightly restricted temporal window of UBR5 action, (ii) functional redundancy and/or (iii) existence of a compensatory/homeostatic mechanism.

Taken together our in vitro and in vivo findings imply that loss of *Ubr5* function correlated with a reduction in *hedgehog* ligand expression and pathway activity. However, we did not establish the molecular mechanism by which UBR5 might influence transcription and/or half-life of *hh*-family mRNAs. A potential explanation resides in the ability of *Drosophila* and mammalian Hyd/UBR5 to bind Sgg/GSK3β [16, 62] and Ci/GLI [16, 63] as well as influence Sgg-mediated *hh* expression [16]. GSK3β’s role as a potent regulator of numerous transcription factors [64], raises the possibility that it may also regulate the activity of known *hedgehog*-regulatory transcription factors such as RUNX2 [65, 66], ATF4 [67], HAND2-TWIST1 [68, 69] and ETV4/5 [70].

While there is currently no evidence to suggest GSK3β regulates *Shh* or *Ihh* expression in mammals, it does bind, phosphorylate and repress GLI proteins [71, 72]. Hyd/UBR5’s ability to bind Sgg/GSK3β and Ci/GLI therefore provides it with the potential to influence Ci/GLI activity downstream of any effects on *Shh/Ihh* ligand expression. Finally, Hyd’s ability to bind chromatin also provides an alternative means to directly regulate gene expression [63]. Interestingly, the ability of one protein to independently regulate *hh* ligand expression and GLI activity is also observed with the Hh-pathway-associated kinase DYRK1B [14]. Together, these observations support the notion that initiation of Hh signalling (ligand production) and its response (GLI activity) can be independently co-ordinated by the action of a single protein.

In the *Ubr5* null embryos, defective Hedgehog signalling may underlie the reported placental vascular defects [73] and embryonic death [48, 53]. Defective placental function may also explain our observed midgestation lethality of *pCAGG-Cre;Ubr5*^mt*/mt*^ embryos, but we cannot exclude an essential role for UBR5 in the embryo proper. The detection of *Ubr5* expression within the pharyngeal arches, a major source for artery development [74] could also provide an alternative/complementary means for UBR5 to influence embryonic vasculature. Our observation that mid-gestational loss of one copy of *Ubr5* resulted in a reduction in the observed/expected progeny, suggested that correct *Ubr5* gene dosage was required for mid-gestation viability. This is in contrast to a lack of any effects on progeny lacking one copy of *Ubr5* from conception. We therefore hypothesise that a gene compensatory mechanism is able to cope with reduced *Ubr5* gene dose from conception, but not in response to an acute decrease in UBR5 function mid-gestation.

The concepts of genetic redundancy and compensation may also explain why we observed no defect in the embryonic *Prx1-Cre;Ubr5*^*mt/mt*^ limbs. A threshold model could also be a potential explanation, whereby only a certain magnitude of change in Hedgehog signalling would elicit a morphological defect. Such a model is strongly supported by the normal limb development of animals with a 50% reduction in *Shh* expression [75]. Furthermore, heterozygous mutant animals of *Shh* [48], *Ihh* [76] and *Smo* [77], also fail to exhibit developmental limb defects. Therefore, the morphological consequences associated with a reduction in, rather than complete loss of, a Hedgehog signalling component varies dramatically.

In summary, the generation and validation of a conditional *Ubr5* mutant mouse provides a useful tool to address murine development and homeostasis. While our findings further highlight the importance of UBR5 in embryonic development and ES cell biology, they also reinforce its potential role in influencing Hedgehog signalling.
